# Divergent strains of EHV-1 in Swedish outbreaks during 2012 to 2021

**DOI:** 10.1186/s12917-024-04096-7

**Published:** 2024-06-22

**Authors:** Johan Öhrmalm, Harindranath Cholleti, Anna-Karin Theelke, Mikael Berg, Gittan Gröndahl

**Affiliations:** 1 Department of Animal Biosciences, Swedish University of Agricultural Sciences, Uppsala, Sweden; 2 Swedish Veterinary Agency, Uppsala, Sweden

**Keywords:** Equine herpes myeloencephalopathy, EHV-1, ORF11, ORF30, ORF34, Phylogeny

## Abstract

*Equid alphaherpesvirus* 1 (EHV-1) is a ubiquitous and significant viral pathogen in horses worldwide, causing a range of conditions, including fever, respiratory disease, abortion in pregnant mares and the severe neurological disease called equine herpes myeloencephalopathy (EHM). Despite that EHV-1 is a notifiable animal disease in Sweden, there is limited knowledge about the circulating strains. This study aimed to analyze the genetic diversity of EHV-1 strains in equine samples from different Swedish outbreaks by partial genome sequencing. Genotyping based on three selected open reading frames ORF11, ORF30, and ORF34 in the viral genome was conducted for 55 outbreaks of EHV-1 spanning from the years 2012 to 2021. The analysis revealed 14 different genovariants, with one prominent genovariant identified in 49% of the outbreaks. Additionally, the study identified seven mutations not previously described. Three new mutations were demonstrated in ORF11, all synonymous, and four new mutations in ORF34, two synonymous, and two non-synonymous. Notably, different EHV-1 genovariants were found in five out of six studied EHM outbreaks, but clonal spreading was shown within the outbreaks. Moreover, the study demonstrated that healthy (recovered) horses that returned from an EHM outbreak at an international meeting in Valencia, Spain (2021), were positive for the virus clone responsible for the severe disease outbreak despite several weeks of quarantine. These findings shed light on the genetic diversity and transmission dynamics of the virus and significantly contribute to better understanding of the epidemiology of EHV-1 in Sweden and globally.

## Introduction

*Equid alphaherpesvirus 1 (EHV-1)* is a globally pervasive and substantial threat to equine populations, causing a range of clinical manifestations, including fever, respiratory disease, abortion in pregnant mares and neurological disease, and the severe neurological condition equine herpes myeloencephalopathy (EHM). The virus’s impact on the equine industry is profound, with prevalence rates ranging from 14.5% to 88% in horse populations worldwide [1–6].

EHV-1 possesses a linear double-stranded DNA genome of 150 kbp, encompassing 80 open reading frames that encode 76 functional proteins [7]. Understanding the genetic makeup of EHV-1 is pivotal for unraveling its evolution, transmission dynamics, and pathogenicity. Currently, 1503 full or partial sequences of the EHV-1 genome are represented in GenBank [8]. During outbreaks, genetic profiles often mirror each other among infected horses, while discrepancies emerge over different time points at the same location [9].

Key reference strains, V592 (from an abortion case) and Ab4 (from a neurological case), serve as benchmarks in understanding EHV-1’s genetic landscape [9]. Notably, a single nucleotide polymorphism in the EHV-1 DNA polymerase gene (ORF30), specifically the substitution from adenosine (A) to guanosine (G) at position 2254, has been proposed as a neuropathogenic marker [9–14]. This G2254/D752 genotype shows a significant association with neurological disease, though variants with the A2254/N752 and C2254/H752 genotypes have also been implicated [15–17].

Studies have reported varying prevalence rates of the G2254/D752 genotype in neurological outbreaks, emphasizing the importance of considering diverse genotypic contributions to EHM [9, 12, 18, 19]. Notwithstanding the increasing prevalence of G2254/D752 strains in the USA [19], there is evidence suggesting that these variants may not exclusively lead to neurological illness, challenging previous assumptions [20–22].

Despite EHV-1 being a notifiable animal infection in Sweden, only abortions and neurological cases were officially reported originally, but respiratory cases were only officially reported after 2021. Notably, passive surveillance during the period 2012–2021 identified 69 outbreaks with abortions and 21 outbreaks with neurological cases [23]. This study aims to address the existing knowledge gaps by analyzing the diversity of EHV-1 strains in equine samples from Swedish outbreaks (2012–2021) through the comparison of selected open reading frames (ORF11, ORF30, ORF34) with other EHV-1 gene sequences available in GenBank. ORF11 and ORF34 were selected since they belong to the most variable sections of the viral genome [24], which is crucial for epidemiological studies. This analysis seeks to enhance our understanding of EHV-1 epidemiology in Sweden, particularly in discerning potential clonal spread patterns among circulating strains.

## Materials and methods

### Animals and sample collection

**Fig. 1:**
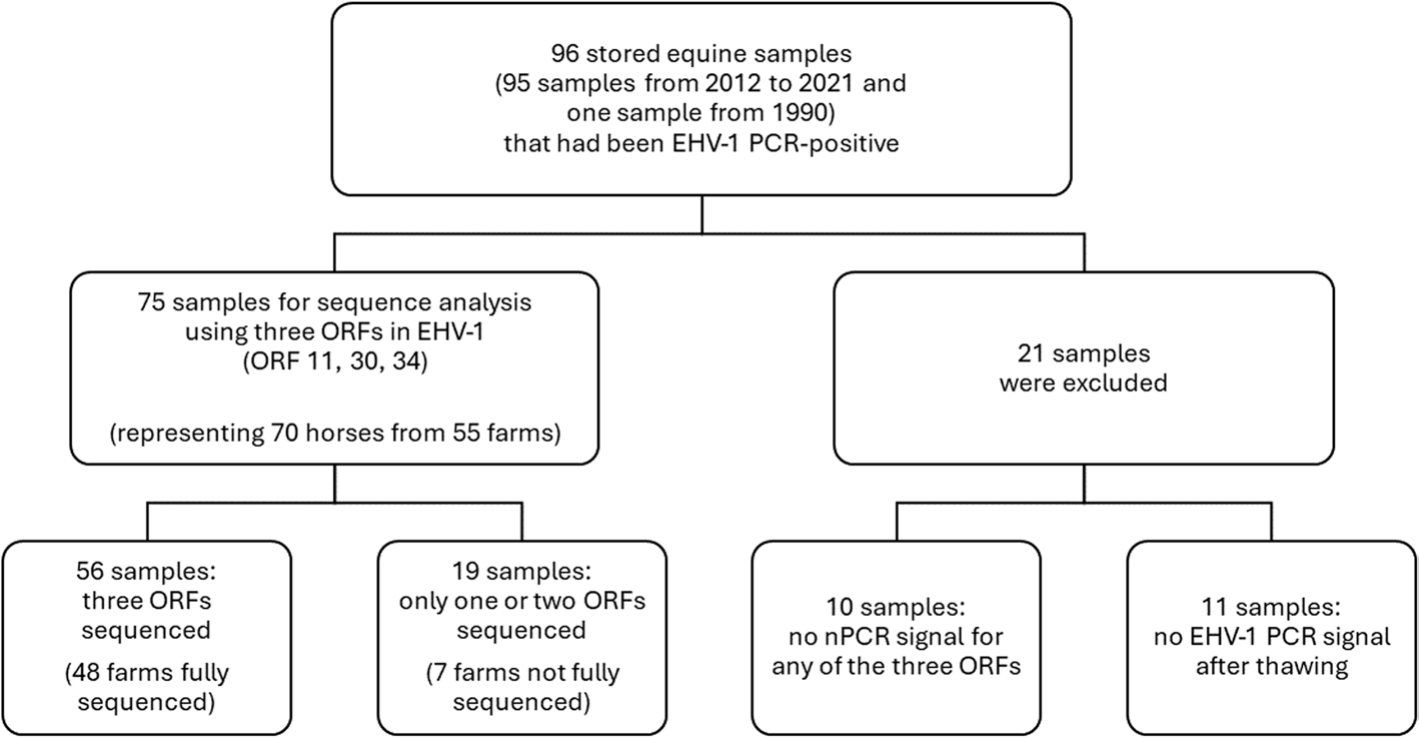
Material for analysis

This retrospective study initially comprised a collection of 96 submitted equine samples that were positive for EHV-1 by RT-PCR [25] at the Swedish Veterinary Agency (SVA) during the 10-year period 2012 to 2021 (*n* = 95), and one sample from 1990 (abortion case, used as laboratory positive control at SVA), all stored at -80 °C. The study material consisted of nasal swabs, blood, fetal organs, or placenta from clinical cases with a history of fever, respiratory signs, neurological signs, or abortion. The study protocol was prepared before the study started. Demographic and clinical data were collected from referral forms, and in some cases incomplete or ambiguous data were amended where possible by contact with the horse owner or the treating veterinarian. Information about EHV-1/4 vaccination status was not available except in one case. Twenty-one samples were excluded (Fig. 1).

The 75 samples that were finally subject to viral genomic analyses represent 70 case horses from 55 different outbreaks, defined as farms with one or more cases of EHV-1 within 2 months (Table 1). Three ORFs in EHV-1 (ORF 11, 30, 34) were successfully sequenced in 56 samples in 48 outbreaks, but in 19 samples in 7 outbreaks, only one or two ORFs could be sequenced (Fig. 1). The samples originated from clinical cases within Sweden, except two samples from returning Swedish horses (SWE/Valencia9G/2021 and SWE/Valencia10E/2021) that had clinically recovered from an outbreak of EHV-1 that occurred in Valencia, Spain in 2021 [26–29], and one case from Norway, 2019. Multiple samples were analyzed from five of the outbreaks (indicated 1 – 5 within curly brackets in Table 1). Two horses were repeatedly sampled at two respectively five time-points for longitudinal follow-up (“3a” and “4b” in Table 1).

**Table 1:** Clinical data for 75 samples from 70 Swedish horses in 55 outbreaks of EHV-1 used in this study

**Strain ID (location/isolate/year)**	**Outbreak no**	**Multiple samples**	**Gender**	**Age (y)**	**Breed**	**Source**	**Disease type**
SWE/1A/1990	1		Mare	ND	ND	Placenta/organs	Abortion
SWE/4H/2011	2		ND	ND	ND	ND	ND
SWE/1H/2012	3		Mare	ND	ND	Placenta/organs	Abortion
SWE/2A/2012	4		Mare	ND	ND	Placenta/organs	Abortion
SWE/4G/2012	5		Mare	ND	ND	Placenta/organs	Abortion
SWE/Upplands-Bro1E/2013	6		Mare	13	STB	Placenta/organs	Abortion
SWE/1F/2013	7		Mare	ND	ND	Placenta/organs	Abortion
SWE/8A/2013	8		Mare	ND	ND	Placenta/organs	Abortion
SWE/8B/2013	9		Mare	ND	ND	Placenta/organs	Abortion
SWE/8C/2013	10		ND	ND	ND	Nose swab	ND
SWE/8D/2013	11		Mare	ND	ND	Placenta/organs	Abortion
SWE/7A/2016	12		Geld	4	WBR	Nose swab	ND
SWE/Upplands Väsby7F/2016	13	{1}	Geld	5	STB	Nose swab	Respiratory
SWE/Upplands Väsby7G/2016	13	{1}	Mare	3	STB	Nose swab	Respiratory
SWE/Halmstad7E/2016	14	{2}	Mare	8	STB	Nose swab	Abort/Neuro
SWE/Halmstad11A/2016	14	{2}	Mare	8	STB	Placenta/organs	Abortion
SWE/Halmstad11B/2016	14	{2}	Mare	9	STB	Placenta/organs	Abortion
SWE/Halmstad11C/2016	14	{2}	Mare	10	STB	Placenta/organs	Abortion
SWE/Sölvesborg6E/2017	15		Mare	ND	ND	Blood	Neuro
SWE/6H/2017	16		Mare	23	WBR	Nose swab	Respiratory
SWE/Heby11D/2017	17		Mare	12	STB	Placenta/organs	Abortion
SWE/Ronneby3C/2018	18		Mare	15	SWB	Blood	Neuro
SWE/8G/2018	19		Mare	19	Icel	Placenta/organs	Abortion
SWE/Varberg11E/2018	20		Mare	ND	ND	Placenta/organs	Abortion
SWE/Sandviken11F/2018	21		Mare	ND	ND	Placenta/organs	Abortion
SWE/Eda11G/2018	22		Mare	ND	STB	Placenta/organs	Abortion
SWE/Haninge12G/2018	23		Mare	ND	Bashkir	Placenta/organs	Abortion
SWE/2C/2019	24		Mare	8	ND	Blood	Respiratory
SWE/Österåker2B/2019	25	{3}(a)	Geld	22	WBR	Blood	Neuro
SWE/Österåker3D/2019	25	{3}(a)	Geld	22	WBR	Nose swab	Neuro
SWE/Österåker4C/2019	25	{3}(a)	Geld	22	WBR	Blood	Neuro
SWE/Österåker4D/2019	25	{3}(a)	Geld	22	WBR	Blood	Neuro
SWE/Österåker4E/2019	25	{3}(a)	Geld	22	WBR	Blood	Neuro
SWE/Österåker2E/2019	25	{3}	Mare	11	Pony	Blood	Neuro
SWE/Österåker3G/2019	25	{3}	Mare	9	Pony	Nose swab	Respiratory
SWE/Österåker3H/2019	25	{3}	Geld	9	Pony	Nose swab	Respiratory
SWE/Österåker4A/2019	25	{3}	Mare	10	Pony	Nose swab	Respiratory
SWE/Österåker4B/2019	25	{3}	Geld	7	WBR	Nose swab	Neuro
SWE/Österåker5A/2019	25	{3}	Mare	14	WBR	Nose swab	Neuro
SWE/Österåker5C/2019	25	{3}	Geld	14	SWB	Nose swab	Neuro
SWE/Österåker7B/2019	25	{3}	Mare	11	Pony	Nose swab	Neuro
SWE/Värmdö3B/2019	26	{4}(b)	Mare	9	Pony	Blood	Neuro
SWE/Värmdö7C/2019	26	{4}(b)	Mare	9	Pony	Nose swab	Neuro
SWE/Värmdö2D/2019	26	{4}	Mare	17	WBR	Blood	Respiratory
SWE/Värmdö5H/2019	26	{4}	Mare	11	Pony	Nose swab	Respiratory
SWE/Skellefteå3E/2019	27		Mare	ND	ND	Placenta/organs	Abortion
SWE/Karlskrona3F/2019	28		Mare	14	ND	Placenta/organs	Abortion
SWE/Norrtälje4F/2019	29		Mare	ND	Arab	Placenta/organs	Abortion
SWE/Älvkarleby5B/2019	30		Geld	11	Pony	Nose swab	Respiratory
SWE/Enköping5D/2019	31		Mare	9	ND	Placenta/organs	Abortion
SWE/Norway5F/2019	32		ND	ND	ND	Nose swab	ND
SWE/Örnsköldsvik5G/2019	33		Mare	9	Bashkir	Placenta/organs	Abortion
SWE/Örebro6A/2019	34		Mare	12	ND	Placenta/organs	Abortion
SWE/Umeå12H/2019	35		Mare	15	ND	Placenta/organs	Abortion
SWE/Strängnäs2G/2020	36		Mare	11	SWB	Placenta/organs	Abortion
SWE/Gotland2H/2020	37		Mare	ND	AQH	Placenta/organs	Abortion
SWE/Huddinge3A/2020	38		Geld	5	WBR	ND	Respiratory
SWE/Ale10C/2020	39		Mare	9	WBR	ND	Respiratory
SWE/Sjöbo11H/2020	40		Mare	11	SWB	Placenta/organs	Abortion
SWE/12D/2020	41		Geld	ND	SWB	Nose swab	Respiratory
SWE/Sala12F/2020	42		Mare	12	SWB	Placenta/organs	Abortion
SWE/Uppsala8H/2021	43		Mare	ND	SWB	Placenta/organs	Abortion
SWE/Hjo9A/2021	44		Mare	17	SWB	Placenta/organs	Abortion
SWE/Hjo9C/2021	45		Mare	ND	ND	Placenta/organs	Abortion
SWE/Kristianstad9H/2021	46		Geld	2	WBR	Nose swab	Respiratory
SWE/Strängnäs10A/2021	47		Mare	4	STB	Nose swab	Respiratory
SWE/Karlsborg10D/2021	48		Mare	ND	SWB	Placenta/organs	Abortion
SWE/Enköping10G/2021	49		Mare	2	STB	Nose swab	Respiratory
SWE/Knivsta10H/2021	50		Mare	ND	STB	Nose swab	Respiratory
SWE/Strängnäs12A/2021	51		Mare	7	Pony	Placenta/organs	Abortion
SWE/Uddevalla12B/2021	52		Mare	11	SWB	Placenta/organs	Abortion
SWE/Boxholm12C/2021	53		Mare	ND	STB	Placenta/organs	Abortion
SWE/12E/2021	54		Mare	ND	ND	ND	Abortion
SWE/Valencia9G/2021	55	{5}	ND	ND	SWB	Nose swab	Neuro
SWE/Valencia10E/2021	55	{5}	ND	ND	ND	Nose swab	Neuro

All samples are from unique horses from unique outbreaks, except for multiple samples from five outbreaks (denotated 1–5)

Letters a and b; repeated samples from two individual horses collected at different occasions

*ND* No data, *Neuro* Neurological signs, *Geld.* Gelding, *y* Years old, *STB* Standardbred, *SWB* Swedish Warmblood riding horse, *WBR* Warmblood riding horse (unknown origin), *AQH* American Quarter horses, *Shetl* Shetland pony, *Icel* Icelandic horse

Gender, age, breed, farm/outbreak assignation, sample source and main clinical finding are presented in Table 1. The demographic data was blinded to the performers of the genetic analysis at the time of the laboratory analysis. The samples were from 55 mares, 10 geldings and 5 horses with no assigned gender, aged 2 – 23 years, and the breeds indicated were Standardbred (*n* = 13), Swedish Warmblood (*n* = 11), warmblood riding horse (undefined type, *n* = 9), pony breed (*n* = 9), Bashkir (*n* = 2), Arabian horse (*n* = 1), American Quarter horse (*n* = 1), Icelandic horse (*n* = 1), or not defined (*n* = 23). In the 55 outbreaks, 6 included at least one horse with neurological signs, 35 included at least one case of abortion, 11 included only respiratory or fever cases, and 4 outbreaks lacked information of clinical signs. One of the outbreaks (Halmstad, 2016) had several cases of abortion, with additional neurological signs in one aborting mare.

### Polymerase Chain Reaction (PCR)

Nucleic acids were extracted using with a IndiMag Pathogen Kit using TANBead Maelstrom 96,000 Nucleic Acid Extractor. Swabs from the nose, blood, and specific organs were collected and placed into TE buffer before nucleic acid extraction.

For flocked nylon swabs in liquid Amies, the liquid was used directly, whereas dry swabs were soaked with buffer and shaken for 15 min. Blood samples was used directly. Tissues were either swabbed, with the swab soaked with buffer and shaken for 15 min, or they were homogenized in buffer. DNA strand separation was done by extraction using streptavidin-coated beads. A Real-Time qPCR in-house assay, TaqMan, targetting glykoprotein C (ORF16, 143 bp) [25] was used to verify detectable levels of virus in the eluates.

PCR amplifications of three Open Reading Frames (ORF) of EHV-1 (ORF11, ORF30, and ORF34) were performed. Nested primer sequences of ORF30 and ORF34 were modified from Preziuso et al. [30] and a new set of primers that target the ORF11 region was designed to amplify a 842 bp amplicon (Table 2).

**Table 2:** Primers for ORF11, ORF30, and ORF34 used in the study

**Gene**	**Primer name**	**Sequence (5´-3´)**	**Product Size (bp)**	**Annealing Temperature**
ORF11	ORF11_F1	CCGATACGTGGCGTAGACG	908	58 °C
	ORF11_R1	ACCGGAATTCGTGTCGTCG		
	ORF11_F2	TGGCTGTAACGATGCTAACG	842	58 °C
	ORF11_R2	TTACATTTCAGGGATCGCCG		
ORF30	F8	GTGGACGGTACCCCGGAC	380	62 °C
	R2	GTGGGGATTCGCGCCCTCACC		
	F7	GGGAGCAAAGGTTCTAGACC	256	62 °C
	R3	AGCCAGTCGCGCAGCAAGATG		
ORF34	1058F	GGCCCCAAGGATATTTAAGC	855	60 °C
	1893R	GTTTGAGGCGGTTACGTCAG		
	1090Fi	CCGAGGTTTCATCCTCATTC	714	60 °C
	1784Ri	GCGGACATATTCGTGTCTCA		

In the PCR reaction, the 25 µl or 50 µl reaction mixture contained 1 × PCR buffer II (10x), 200 µM of each dNTP, 15 mM MgCl_2_, 300 µM of each primer, 2.5 µl of DNA template from the eluate and 2.5 U or 5 U AmpliTaq Gold DNA polymerase (Applied Biosystems, Foster City, CA, USA). After evaluating initial PCR reactions, the annealing temperatures of ORF30 and ORF34 primers were adjusted to +2 degrees Celsius higher compared to Preziuso et al. [30]. PCR conditions were 95 °C for 10 min, 35 cycles of denaturation at 95 °C for 15 s, annealing temperature 58–62 °C for 30 s, extension at 72 °C for 1 min, and a final extension at 72 °C for 5 min. PCR products were visualized on 1.2 – 2% agarose gel. Samples with no or weak band on the first PCR were subject to nPCR. The amplicons with expected size, including the cut out of DNA bands from agarose gels, were cleaned with GeneJet Gel Extraction Kit (Thermo Fisher Scientific, Waltham, MA, USA). Eluates were sent to Macrogen Europe B.V. (Netherlands) for Sanger sequencing.

A real-time PCR analysis for differentiation of A2254 and G2254 polymorphism in ORF30 performed according to Smith et al. [31] was used for two samples (SWE/Älvkarleby5B/2019 and SWE/1A/1990; Table 3), in which the sequencing of ORF30 was not fully successful. A minor modification of the described method [31] was the use of VIC-MGB (Minor Groove Binder) in the probe, instead of HEX-MGB.

**Table 3:** Results from sequencing of ORF11, ORF30, and ORF34 in samples from 55 Swedish EHV-1 outbreaks

			ORF and DNA position																	
			ORF11 129	ORF11 141^a^	ORF11 156	ORF11 390^a^	ORF11 704	ORF11 713	ORF11 748	ORF11 765^a^	ORF30 2225	ORF30 2254	ORF34 136^a^	ORF34 148	ORF34 156	ORF34 282	ORF34 303	ORF34 309^a^	ORF34 408^a^	ORF34 434^a^
Samples from Outbreaks	Genovariant	*Ab4*	*A*	*C*	*T*	*A*	*G*	*A*	*G*	*G*	*T*	*G*	*G*	*A*	*G*	*T*	*C*	*G*	T	C
SWE/1H/2012	1		·	·	·	·	·	·	·	·	·	A^b^	·	·	·	·	·	·	·	·
SWE/2A/2012	1		·	·	·	·	·	·	·	·	·	A^b^	·	·	·	·	·	·	·	·
SWE/4G/2012	1		·	·	·	·	·	·	·	·	·	A^b^	·	·	·	·	·	·	·	·
SWE/8A/2013	1		·	·	·	·	·	·	·	·	·	A^b^	·	·	·	·	·	·	·	·
SWE/8B/2013	1		·	·	·	·	·	·	·	·	·	A^b^	·	·	·	·	·	·	·	·
SWE/7A/2016	1		·	·	·	·	·	·	·	·	·	A^b^	·	·	·	·	·	·	·	·
SWE/6H/2017	1		·	·	·	·	·	·	·	·	·	A^b^	·	·	·	·	·	·	·	·
SWE/Heby11D/2017	1		·	·	·	·	·	·	·	·	·	A^b^	·	·	·	·	·	·	·	·
**SWE/Ronneby3C/2018**	1		·	·	·	·	·	·	·	·	·	A^b^	·	·	·	·	·	·	·	·
SWE/8G/2018	1		·	·	·	·	·	·	·	·	·	A^b^	·	·	·	·	·	·	·	·
SWE/Varberg11E/2018	1		·	·	·	·	·	·	·	·	·	A^b^	·	·	·	·	·	·	·	·
SWE/Sandviken11F/2018	1		·	·	·	·	·	·	·	·	·	A^b^	·	·	·	·	·	·	·	·
SWE/2C/2019	1		·	·	·	·	·	·	·	·	·	A^b^	·	·	·	·	·	·	·	·
SWE/Karlskrona3F/2019	1		·	·	·	·	·	·	·	·	·	A^b^	·	·	·	·	·	·	·	·
SWE/Örnsköldsvik5G/2019	1		·	·	·	·	·	·	·	·	·	A^b^	·	·	·	·	·	·	·	·
SWE/Huddinge3A/2020	1		·	·	·	·	·	·	·	·	·	A^b^	·	·	·	·	·	·	·	·
SWE/Sjöbo11H/2020	1		·	·	·	·	·	·	·	·	·	A^b^	·	·	·	·	·	·	·	·
SWE/Sala12F/2020	1		·	·	·	·	·	·	·	·	·	A^b^	·	·	·	·	·	·	·	·
SWE/Uppsala8H/2021	1		·	·	·	·	·	·	·	·	·	A^b^	·	·	·	·	·	·	·	·
SWE/Hjo9A/2021	1		·	·	·	·	·	·	·	·	·	A^b^	·	·	·	·	·	·	·	·
SWE/Hjo9C/2021	1		·	·	·	·	·	·	·	·	·	A^b^	·	·	·	·	·	·	·	·
SWE/Strängnäs10A/2021	1		·	·	·	·	·	·	·	·	·	A^b^	·	·	·	·	·	·	·	·
SWE/Enköping10G/2021	1		·	·	·	·	·	·	·	·	·	A^b^	·	·	·	·	·	·	·	·
SWE/Knivsta10H/2021	1		·	·	·	·	·	·	·	·	·	A^b^	·	·	·	·	·	·	·	·
SWE/Uddevalla12B/2021	1		·	·	·	·	·	·	·	·	·	A^b^	·	·	·	·	·	·	·	·
SWE/Boxholm12C/2021	1		·	·	·	·	·	·	·	·	·	A^b^	·	·	·	·	·	·	·	·
SWE/12E/2021	1		·	·	·	·	·	·	·	·	·	A^b^	·	·	·	·	·	·	·	·
SWE/8C/2013	2		·	·	C	·	T^b^	·	·	·	·	A^b^	·	·	·	·	·	·	·	·
SWE/8D/2013	2		·	·	C	·	T^b^	·	·	·	·	A^b^	·	·	·	·	·	·	·	·
SWE/Norway5F/2019	2		·	·	C	·	T^b^	·	·	·	·	A^b^	·	·	·	·	·	·	·	·
SWE/Ale10C/2020	2		·	·	C	·	T^b^	·	·	·	·	A^b^	·	·	·	·	·	·	·	·
SWE/12D/2020	2		·	·	C	·	T^b^	·	·	·	·	A^b^	·	·	·	·	·	·	·	·
SWE/Kristianstad9H/2021	2		·	·	C	·	T^b^	·	·	·	·	A^b^	·	·	·	·	·	·	·	·
**SWE/Värmdö2D/2019**	3		·	·	C	·	T^b^	·	·	·	·	G	·	·	·	·	·	·	·	·
SWE/Norrtälje4F/2019	3		·	·	C	·	T^b^	·	·	·	·	G	·	·	·	·	·	·	·	·
**SWE/Sölvesborg6E/2017**	4		·	·	·	·	·	·	·	·	·	G	A^b^	·	·	·	·	·	·	·
**SWE/Österåker2E/2019**	4		·	·	·	·	·	·	·	·	·	G	A^b^	·	·	·	·	·	·	·
SWE/Strängnäs12A/2021	5		·	·	·	·	·	·	·	·	C^b^	A^b^	·	·	·	·	·	·	·	·
SWE/Strängnäs2G/2020	6		G	·	C	·	T^b^	·	·	·	·	A^b^	·	·	·	·	·	·	·	·
SWE/Karlsborg10D/2021	7		·	·	·	·	·	·	·	·	·	A^b^	·	·	·	·	·	·	·	A^b^
**SWE/Valencia10E/2021**	8		·	·	·	·	·	G^b^	·	·	·	A^b^	·	·	·	·	·	·	·	·
SWE/Umeå12H/2019	9		·	·	·	G	·	·	·	·	·	A^b^	·	·	·	·	·	·	·	·
SWE/UpplandsVäsby7F/2016	10		·	·	C	·	·	·	·	·	·	G	·	·	·	·	·	·	·	·
**SWE/Halmstad11A/2016**	10		·	·	C	·	·	·	·	·	·	G	·	·	·	·	·	·	·	·
SWE/Haninge12G/2018	11		-	T	C	·	·	·	·	·	·	G	·	·	·	·	·	A	·	·
SWE/1F/2013	12		·	·	·	·	·	·	T^b^	·	·	G	·	·	T^b^	·	A	·	·	·
SWE/Skellefteå3E/2019	13		·	·	·	·	·	·	T^b^	A	·	A^b^	·	·	T^b^	·	A	·	·	·
SWE/Eda11G/2018	14		·	·	·	·	·	·	T^b^	·	·	A^b^	·	·	T^b^	·	A	·	·	·
SWE/Älvkarleby5B/2019	-		·	·	·	·	·	·	·	·	-	A^b,c^	·	·	·	·	·	·	C	·
SWE/Gotland2H/2020	-		-	-	-	-	-	-	-	-	-	-	·	·	·	·	·	·	·	·
SWE/Enköping5D/2019	-		-	-	-	-	-	-	-	-	·	A^b^	·	·	·	·	·	·	·	·
SWE/Örebro6A/2019	-		-	-	-	-	-	-	-	-	·	A^b^	-	-	-	-	-	-	-	-
SWE/1A/1990	-		-	-	-	-	-	-	-	-	-	G^c^	·	G^b^	·	C	·	·	·	·
SWE/Upplands-Bro1E/2013	-		-	-	-	-	-	-	-	-	·	G	·		T^b^	·	A	·	·	·
SWE/4H/2011	-		-	-	-	-	-	-	-	-	·	G	·	·	·	·	·	·	·	·

Outbreaks with neurological cases are marked bolded

- Lack of data (not possible to sequence). Bases of reference strain Ab4 is shown for each ORF and DNA position where a mutation is detected in an outbreak strain. For each outbreak, DNA mutations are shown in the matrix

A dot indicate no change compared to Ab4

^a^Positions for mutations that were not reported before (according to NCBI, 2021)

^b^Mutations that cause changes in amino acids

^c^Result from PCR analysis for differentiation of A2254 and G2254 polymorphism

### Bioinformatic analysis

The sequences were edited using Unipro UGENE (version 40.1). The two sequences for each sample were trimmed and compared with BLAST database and the reference sequence EHV-1 Ab4 (GenBank accession number AY665713.1). The characterized sequences were deposited in GenBank (Accession numbers OR941084-OR941125). The sequences were compared with other EHV-1 sequences from the GenBank to identify mutations, by multiple sequence analysis performed on MEGAX-11.0.8 with the ClustalW plugin.

To compare the strains and construct a concatenated sequence, partial genome sequencing was used by cutting the trimmed sequences at the same position in both 5’ and 3’ ends for each ORF; for ORF11, 698 bases in position 125–822; ORF30, 135 bases in position 2165–2298, and for ORF34, all 483 bases position 1–483. The cut sequences, for each strain, could then be concatenated for ORF11, ORF30, and ORF34, in that order. Phylogenetic trees were generated using the likelihood model, which was applied to concatenated sequences, and this analysis included 1000 bootstrap replicates.

### Statistical analysis

Prevalence of genetic variants in different clinical groups were analyzed with Fischer’s exact test (openepi.com) and *p* < 0.05 was considered significant.

## Results and discussion

This study encompasses 55 Swedish EHV-1 outbreaks over a decade. Of these outbreaks, 11% involved neurological cases, at least 64% involved abortions, 20% involved only respiratory cases, and 7% had an unknown history. The study covered over half of the 90 officially recorded cases of abortion or neurological disease caused by EHV-1 in Sweden during this period, the forms of the disease that were notifiable. However, there was a lack of surveillance data for outbreaks that only presented with fever or respiratory disease, as these were not notifiable at the time; nonetheless, the study included data from at least 11 such outbreaks. Consequently, the study material is deemed representative, providing a comprehensive overview of the diversity of EHV-1 strains circulating in Sweden from 2012 to 2021.

**Fig. 2:**
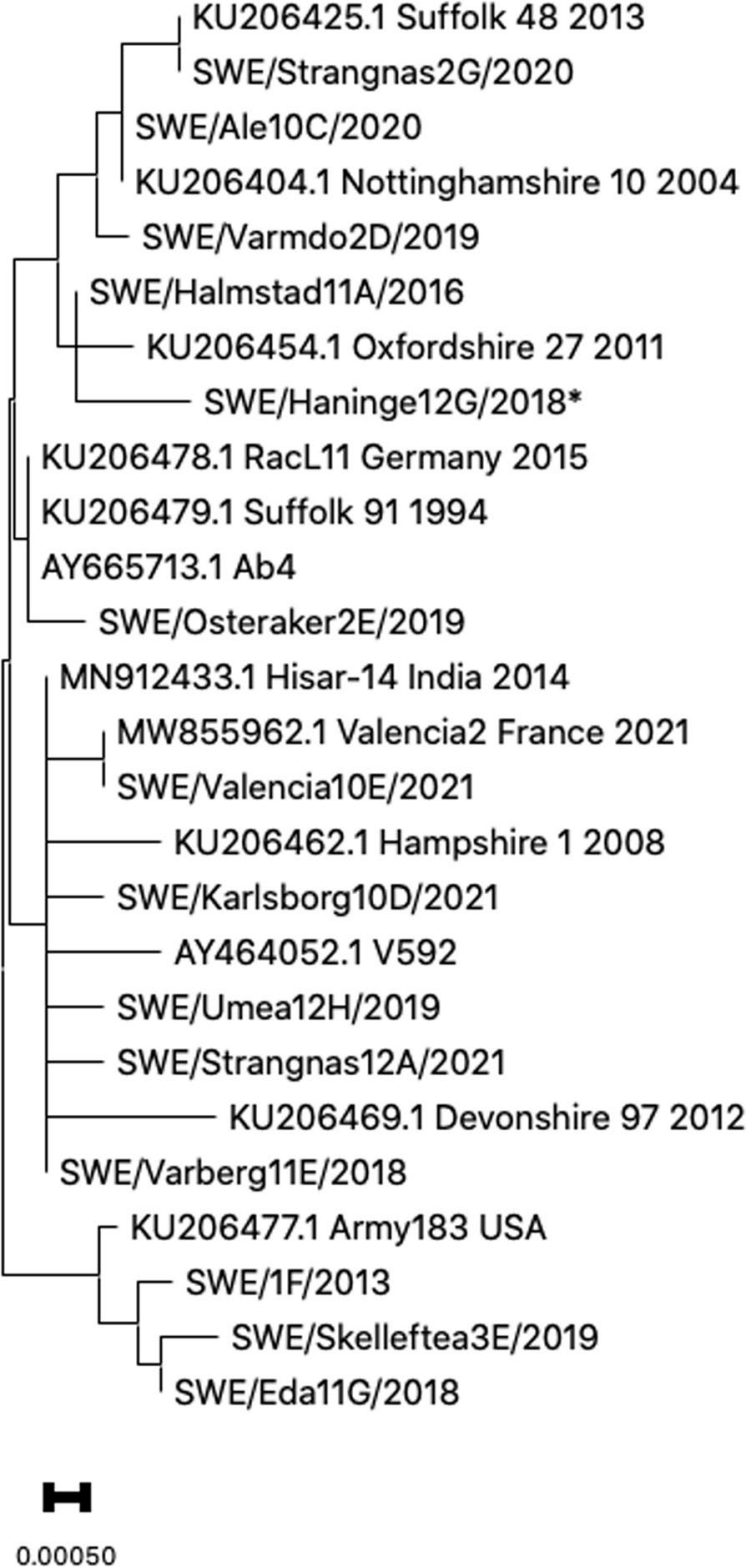
Phylogenic tree for concatenated form of ORF11, ORF30, and ORF34 from 49 Swedish outbreaks

The findings from sequence analysis and genotyping using ORF11, ORF30, and ORF34 for one representative sample from each of the 55 outbreaks (premises), are presented in Table 3, with comparison to reference strain Ab4. Amino acid differences are detailed in Table 4, and a concatenated tree of ORF11, ORF30, and ORF34 genotypes is illustrated in Fig. 2. Notably, multiple samples from five outbreaks showed identical sequencing results within each outbreak, contributing to the robustness of the study. In total, 18 mutations, including ORF30 G2254A, were identified, with seven mutations previously unreported (Table 3). Out of 18 mutations, nine mutations were non-synonymous, causing amino acid changes (Table 4). A limitation of the study is that whole genome sequencing was not available, which would have increased the resolution of the genotyping.

**Table 4:** Mutations and amino acid shifts in genes ORF11, ORF30, and ORF34 found in this study

**Gene**	**Mutation**	**Reference**	**Amino acid shift**
ORF11	A129G	[24]	-
	C141T	This study	-
	T156C	[32]	-
	A390G	This study	-
	G704T	[32]	R235M
	A713G	[28]	K238R
	G748T	[33]	A250S
	G765A	This study	-
ORF30	T2225A	[34]	V742A
	G2254A	[12]	D752N
ORF34	G136A	This study	D46N
	A148G	[35]	T50A
	G156T	[36]	Q52H
	T282C	[35]	-
	C303A	[37]	-
	G309A	This study	-
	T408C	This study	-
	C434A	This study	P145Q

Interpretation of mutation: First letter indicates nucleotide in reference strain Ab4, the number is the position, and last letter the mutation. ‘This study’ in the reference column marks mutations discovered in this study. Interpretation of amino acid: First letter indicates amino acid in reference strain Ab4, the number is the position, and last letter the new amino acid. Synonymous mutations are marked with ‘-’

Among the 54 EHV-1 outbreaks with results for ORF30, 43 had the A2254/N752 genotype (80%), and 11 had the G2254/D752 genotype (20%), while the C2254/H752 genotype, which has been described in studies from France and the USA [15–17], was not observed. The predominance of A2254/N752 is in accordance with recent studies of 257 American EHV-1 cases collected from 2019 to 2022; however, they also reported 3.1% with C2254/H752 [17].

Outbreaks with neurological manifestations were more often associated with the ORF30 G2254 variant (4 of 6; 66.7%) than outbreaks involving only abortions or respiratory cases (6 of 47; 12.8%) (Table 3; *p* = 0.0087). This is in concordance with previous studies where the G2254 variant was implicated as a neuropathogenic marker [9, 11–14].

Among the four G2254 strains identified in neurological outbreaks, at least three exhibited varying sequences within ORF11 and/or ORF34, signifying that they were not the same clone but distinct strains. Interestingly, the study unveiled that the two simultaneous large outbreaks of EHM in Värmdö and Österåker in 2019 with close temporal and spatial proximity (18 km linear distance) were caused by two separate genovariants of EHV-1 (3 and 4, Table 3). However, the Värmdö EHM outbreak in 2019 shared genovariant 3 with an abortion case in Norrtälje (outbreak 29) in the same region (35 km linear distance) and the same year. Additionally, the Österåker outbreak in 2019 shared genovariant 4 with an earlier neurological case in another region, Sölvesborg, in 2017. Despite the similar genovariants, clear epidemiological links could not be established in either of these instances.

The ORF30 A2254 variant was detected in two out of six neurological outbreaks: one in Ronneby (south of Sweden) in 2018, and the other involving Swedish horses from the EHV-1 neurological disease outbreak in Valencia, Spain, in 2021 [26–28]. Despite sharing the same ORF30 variant, these two outbreaks were caused by different strains, as indicated by patterns in ORF11. The partial sequencing results from Swedish Valencia cases were consistent with the whole genome sequences reported in Belgian, French and Swiss horses linked to the Valencia outbreak [28, 29], supporting the notion of the spread of a single EHV-1 strain during the 2021 neurological disease outbreak in Spain. Another instance of strains with similar genotypes, suggesting potential clonal spread, occurred in two separate Swedish EHM outbreaks in 2019 (outbreaks 25 and 26 in Table 1), indicating likely transmission among resident horses. However, clonal spread between premises of EHV-1 strains causing EHM has not been the general case in Sweden, since five out of six EHV-1 outbreaks involving neurological cases in our study were clearly caused by different strains (Table 3).

In ORF30, a non-synonymous mutation in position 2225 causing an amino acid shift from valine (V) to alanine (A) (V742A) was identified in one aborting mare (SWE/Strängnäs12A/2021; Table 4). This mutation, previously reported by Yamada et al. [34], was also observed in another Swedish premise in 2021, not included in this study (personal communication, H. J. Nauwynck, Belgium). Our tracing revealed a common contact point for these two Swedish case horses from different outbreaks in 2021, which might constitute a transmission event.

In ORF34, our investigations revealed eight mutations of which four mutations were novel, see Tables 3 and 4. Two out of the four new mutations were non-synonymous, resulting in amino acid alterations (Table 4).

Within ORF11, eight mutations were identified (Table 3). Notably, three of these mutations, all synonymous, had not been reported previously (Table 4).

Through the combination of results from ORF11, ORF30, and ORF34, genotyping was achieved for 49 outbreaks, identifying 14 distinct genovariants numbered 1 to 14, none of which were identical to Ab4 (see Table 3). The most prevalent genotype was genovariant 1 (*n* = 27), followed by genovariant 2 (*n* = 6). Genovariants 3, 4 and 10 were found in two outbreaks each, while the remaining ten genovariants were observed in single outbreaks.

Unique mutations that were not reported before were identified in two isolates, SWE/Haninge12G/2018 (genovariant 11) and SWE/Älvkarleby5B/2019, adding to the diversity of EHV-1. Eleven genovariants have mutations in none or only one of ORF11 and ORF34 showing that both these ORF’s are of great importance to identify strains. The isolates in six outbreaks were not assigned a genovariant based on the combination of ORF11, ORF30, and ORF34, since only one or two ORF’s could be sequenced (Table 3).

Genovariants based on combining three ORF’s, as described in this study, offer higher resolution compared to methods that group based on single ORF’s, such as those used in the study by Preziuso et al. [30]. However, they provide lower resolution than methods that involve sequencing larger parts of the genome, as demonstrated by Bryant et al. [24].

A concatenated phylogenetic tree, encompassing ORF11, ORF30, and ORF34, is presented in Fig. 2. One representative strain from each of the genovariants 1 to 14 among 49 Swedish outbreaks between 2012 and 2021 is shown in the tree, along with the reference strains Ab4 and V592, as well as ten international strains of EHV-1 from GenBank, spanning the years 1994 to 2021. SWE/Haninge12G/2018 lacks data for position 129 in ORF11. To hypothesize where this sample might fit in the phylogenetic tree, we have, for argument’s sake, assumed it has an ‘A’ at position 129, the most common nucleotide. A ‘G’ in this position would generate the same phylogenetic tree, except that SWE/Haninge12G/2018 would be slightly further away from SWE/Halmstad11A/2016 (genovariant 10).

Despite the discovery of new mutations, Swedish strains do not form a distinct group but rather align with variants described in other countries (Fig. 2). It is not surprising, given that Swedish horses actively participate in international equine sports and approximately 10,000 horses enter Sweden annually for various purposes. With increasingly available technology, it is expected that analysis of more strain collections from various regions around the world will add to the understanding of the diversity of the global pool of EHV-1 and the generalizability of regional findings. Our observations suggest that the extensive global trade and movement of horses facilitate both direct and indirect transmission of respiratory pathogens, which contributes to the intricate interplay of viral strains on a global scale.

## Conclusions

In conclusion, this study represents the first comprehensive exploration of the genetic diversity of EHV-1 in Sweden, a country renowned for its vibrant equestrian activity both nationally and internationally. The decade-long dataset encompassing 55 outbreaks contributes novel insights into circulating genovariants of EHV-1. This information, coupled with thorough outbreak investigations, enhances our comprehension of the disease, and facilitates the formation of effective preventative and mitigative measures.

Notably, the study revealed a striking contrast to local perceptions in a region experiencing two large temporally and spatially clustered outbreaks of EHM. The demonstration that these outbreaks were caused by different EHV-1 strains contradicted common local beliefs and underscores that multiple EHV-1 infections may cluster temporally. This highlights the need for robust infection surveillance and control strategies. The collection of Swedish EHV-1 strains showed significant diversity, contributing new variants to the global gene bank. Despite one genovariant dominating nearly half of the 55 outbreaks, the identification of 13 additional genovariants clustering with viruses isolated worldwide emphasizes the inherent risk of disease transmission through the extensive global connections within the equine industry.

A noteworthy example, showing this global interconnectedness, is the identification of asymptomatic horses returning from an EHM outbreak in an equestrian show in Valencia, Spain, still carrying the specific strain even after weeks of quarantine. Of particular interest is also the finding that, among the six studied outbreaks with neurological cases, five were attributed to different EHV-1 genovariants. While the G2254/D752 genotype exhibited a significant association with neurological outbreaks, it failed to explain all the variety in clinical manifestations.

These observations underscore the complexity of EHV-1 transmission dynamics. The study’s findings also highlight the challenges in establishing direct epidemiological links based solely on genetic information, emphasizing the need for a multifaceted approach integrating both molecular and epidemiological data to unravel the intricate dynamics of EHV-1 outbreaks.

## Data Availability

The datasets supporting the conclusions of this article are included within the article, in GenBank, or are available through contact with the corresponding author.

## Abbreviations

Ab4: EHV-1 reference strain, neurological case
bp: Base pair
DNA: Deoxyribonucleic acid
EHV-1: Equid herpesvirus 1
EHM: Equine herpes myeloencephalopathy
kbp: Kilo base pair
nPCR: Nested polymerase chain reaction
ORF: Open Reading Frame
ORF11: Open Reading Frame 11
ORF30: Open Reading Frame 30
ORF34: Open Reading Frame 34
PCR: Polymerase chain reaction
RT-PCR: Real-time polymerase chain reaction
V592: EHV-1 reference strain, abortion case
